# Localized FCM Clustering with Spatial Information for Medical Image Segmentation and Bias Field Estimation

**DOI:** 10.1155/2013/930301

**Published:** 2013-07-16

**Authors:** Wenchao Cui, Yi Wang, Yangyu Fan, Yan Feng, Tao Lei

**Affiliations:** ^1^School of Electronics and Information, Northwestern Polytechnical University, Xi'an 710072, China; ^2^College of Science, China Three Gorges University, Yichang 443002, China

## Abstract

This paper presents a novel fuzzy energy minimization method for simultaneous segmentation and bias field estimation of medical images. We first define an objective function based on a localized fuzzy *c*-means (FCM) clustering for the image intensities in a neighborhood around each point. Then, this objective function is integrated with respect to the neighborhood center over the entire image domain to formulate a global fuzzy energy, which depends on membership functions, a bias field that accounts for the intensity inhomogeneity, and the constants that approximate the true intensities of the corresponding tissues. Therefore, segmentation and bias field estimation are simultaneously achieved by minimizing the global fuzzy energy. Besides, to reduce the impact of noise, the proposed algorithm incorporates spatial information into the membership function using the spatial function which is the summation of the membership functions in the neighborhood of each pixel under consideration. Experimental results on synthetic and real images are given to demonstrate the desirable performance of the proposed algorithm.

## 1. Introduction

Medical image segmentation plays an important role in a variety of biomedical-imaging applications, such as the quantification of tissue volumes, diagnosis, localization of pathology, study of anatomical structure, treatment planning, and computer-integrated surgery [1]. However, segmentation of medical images involves three main image-related problems [2]. First, images contain noise that can alter the intensity of a pixel such that its classification becomes uncertain. Second, images exhibit intensity inhomogeneity where the intensity level of a single tissue class varies gradually over the extent of the image. Third, images have finite pixel size and are subject to partial volume averaging where individual pixel volumes contain a mixture of tissue classes so that the intensity of a pixel in the image may not be consistent with any one class. To overcome these problems, many segmentation techniques have been proposed in the past decades, such as the expectation maximization (EM) algorithm [3–5], level set method [6–9], clustering [10–17], and so on.

Clustering for image segmentation usually classifies image pixels into *c*-clusters such that members of the same cluster are more similar to one another than to members of other clusters, where the number, *c*, of clusters is usually predefined or set by some validity criterion or a priori knowledge [18]. In the clustering methods, fuzzy *c*-means (FCM) based algorithms have been widely used in medical image segmentation. Such a success chiefly attributes to the introduction of fuzziness for the belongingness of each image pixel. This enables the clustering methods to retain more information from the original image than the crisp or hard segmentation methods [10].

Pham and Prince proposed an adaptive FCM algorithm [10] and its extension to 3D data [11], which incorporated a spatial penalty term into the objective function to enable the estimated membership functions to be spatially smoothed. Ahmed et al. [12] modified the objective function of the standard FCM algorithm to compensate for intensity inhomogeneity and to allow the labeling of a pixel to be influenced by the labels in its immediate neighborhood. Liew and Yan [13] used a B-spline surface to model the bias field and incorporated the spatial continuity constraints into fuzzy clustering algorithm. Zhang and Chen [14] replaced the original Euclidean distance with a kernel-induced distance and supplemented the objective function with a spatial penalty term, which modeled the spatial continuity compensation. Incorporating spatial information into the membership function, Chuang et al. [15] proposed a modified FCM algorithm which was less sensitive to noise and yielded more homogeneous segmented regions. L. Szilágri et al. [16] proposed an efficient FCM clustering model for compensating intensity inhomogeneity and segmentation of magnetic resonance (MR) images, which drastically reduced the processing time without causing relevant change in terms of accuracy. Recently, local intensity information has been taken into account to deal with intensity inhomogeneity in fuzzy segmentation method. For example, Li et al. [17] proposed a new fuzzy energy minimization method based on coherent local intensity clustering (CLIC) for simultaneous tissue classification and bias field estimation of MR images. CLIC algorithm draws upon intensity information in local regions; therefore, it can be used to segment images with intensity inhomogeneity. However, spatial information is not taken into account in the CLIC algorithm; as a result, the CLIC algorithm is sensitive to noise.

Our proposed algorithm in this paper is motivated by the localized *K*-means clustering model proposed by Chen et al. in [6]. By introducing the fuzzy belongingness of each pixel into Chen's model, we develop a localized FCM algorithm for image segmentation. We define a fuzzy energy that depends on membership functions, a bias field that accounts for the intensity inhomogeneity, and the constants that approximate the true intensities of the corresponding tissues. Hence, image segmentation and bias field estimation are simultaneously achieved as the result of minimizing this energy. Besides, we incorporate spatial information into the membership function to suppress noise. As an important application, our proposed algorithm can effectively segment medical images with intensity inhomogeneity and noise.

The remainder of this paper is organized as follows.  Section 2 reviews a relevant method. In Section 3, we represent the definition and minimization of the proposed fuzzy energy in detail. We describe how to utilize neighborhood spatial information in Section 4. The algorithm implementation and experimental results are given in Section 5. The discussion on the setting of important parameters is given in Section 6. We end this paper by the conclusion in Section 7.

## 2. Background

Chen et al. [6] applied a localized *K*-means clustering to form an objective function modeling the problem of segmentation and bias field estimation for brain MR images. The image model of intensity inhomogeneity they used is defined as

(1) $$\begin{array}{c} \log I=\log J+\log b, \end{array}$$

where *I* is the measured image intensity, *J* is the true image to be restored, and *b* is an unknown bias field. Let $\tilde{I}$, $\tilde{J}$, and $\tilde{b}$ represent log⁡*I*, log⁡*J*, and log⁡*b*, respectively, then (1) can be rewritten as

(2) $$\begin{array}{c} \tilde{I}=\tilde{J}+\tilde{b}. \end{array}$$

A generally accepted assumption on the bias field $\tilde{b}$ is that it is smooth or slowly varying [19]. Ideally, the intensity $\tilde{J}$ belonging to the *i*th tissue should take a specific value *c*_*i*_, which represents the measured physical property [6, 8, 17].

**Figure 1:**
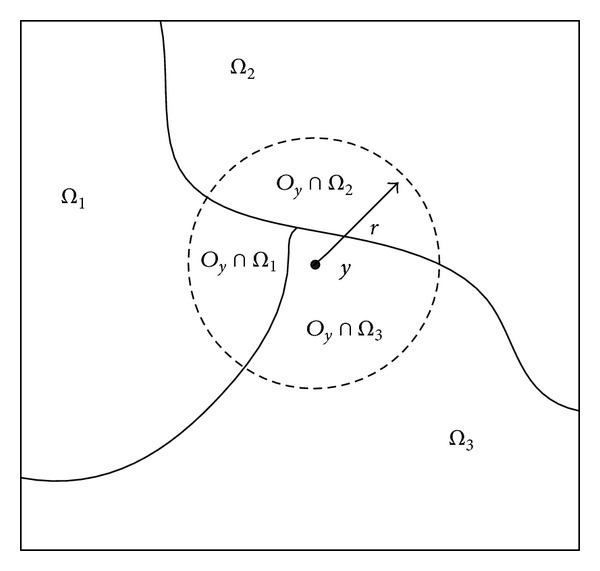
Graphical representation of *O*_*y*_∩*Ω*_*i*_. The dashed circle denotes the circular neighborhood *O*_*y*_ centered on *y*. The image domain *Ω* is divided into three disjoint regions *Ω*_1_, *Ω*_2_, and *Ω*_3_, which partition the neighborhood *O*_*y*_ into three subregions *O*_*y*_∩*Ω*_1_, *O*_*y*_∩*Ω*_2_, and *O*_*y*_∩*Ω*_3_.

Chen's method is based on an observation that pixel intensities in a relatively small region are separable. Let *O*_*y*_ = {*x* : |*x* − *y*| ≤ *r*} denote a circular neighborhood with a relatively small radius *r* centered on each point *y* in the image domain *Ω*. The partition {*Ω*_*i*_}_*i*=1_^*N*^  (*N* is the total number of segmented regions) of the entire domain *Ω* induces a partition of the neighborhood *O*_*y*_; that is, {*O*_*y*_∩*Ω*_*i*_}_*i*=1_^*N*^  forms a partition of *O*_*y*_. For example, Figure 1 presents an image consisting of three disjoint regions: *Ω*_1_, *Ω*_2_, and *Ω*_3_, which divide the neighborhood *O*_*y*_ into three subregions: *O*_*y*_∩*Ω*_1_, *O*_*y*_∩*Ω*_2_, and *O*_*y*_∩*Ω*_3_. Chen et al. defined an objective function to classify the data $\tilde{I}(x)$ in the neighborhood *O*_*y*_ into *N* clusters using a *K*-means clustering method:

(3) $$\begin{array}{c} \xi_{y}=\sum_{i=1}^{N}\int_{O_{y}\cap \Omega_{i}}^{}\omega \left(x-y\right){\left|\tilde{I}\left(x\right)-\tilde{b}\left(y\right)-c_{i}\right|}^{2}dx, \end{array}$$

where $\tilde{b}(y)$ is the value of bias field $\tilde{b}$ at the center of *O*_*y*_, which is approximately equal to the value $\tilde{b}(x)$ for all *x* ∈ *O*_*y*_ on account of the smoothness of the bias field [6]; that is

(4) $$\begin{array}{c} \tilde{b}\left(x\right)\approx \tilde{b}\left(y\right),\;x\in O_{y}. \end{array}$$

Thus, $\left(\tilde{b}(y)+c_{i}\right)$  (*i* = 1,…, *N*) are considered as the approximations of the cluster centers within the neighborhood *O*_*y*_, and *ω*(*x* − *y*) is a nonnegative weighting function such that *ω*(*x* − *y*) = 0 for |*x* − *y* | >*r* and ∫_*O*_*y*__ *ω*(*x* − *y*)*dx* = 1. Note that for each point *y*,  *ω*(*x* − *y*) has the nonzero value with respect to *x* only in *x* ∈ *O*_*y*_. Therefore, (3) can be rewritten as

(5) $$\begin{array}{c} \xi_{y}=\sum_{i=1}^{N}\int_{\;\Omega_{i}}\omega \left(x-y\right){\left|\tilde{I}\left(x\right)-\tilde{b}\left(y\right)-c_{i}\right|}^{2}dx. \end{array}$$

The ultimate goal is to find an optimal set of partitions for the entire image domain *Ω*, the bias field $\tilde{b}$, and the constants *c*_*i*_. The minimization of a single criterion *ξ*_*y*_ for a point *y* does not accomplish this goal. The method minimizes *ε*_*y*_ for all *y* ∈ *Ω*. This can be achieved by minimizing the integral of *ξ*_*y*_ over *Ω*. Therefore, the energy is written as

(6) $$\begin{array}{c} \xi =\int_{\Omega }\left(\sum_{i=1}^{N}\int_{\;\Omega_{i}}\omega \left(x-y\right){\left|\tilde{I}\left(x\right)-\tilde{b}\left(y\right)-c_{i}\right|}^{2}dx\right)dy. \end{array}$$

The above energy *ξ* is expressed in terms of the regions *Ω*_1_,…, *Ω*_*N*_. It is difficult to derive a solution to the energy minimization problem from this expression of *ξ*. Alternatively, we can use one or multiple level set functions to represent the disjoint regions *Ω*_1_,…, *Ω*_*N*_ as in [20]. Thus, this energy *ξ* can be converted into an equivalent level set formulation, which can be solved by using well-established variational methods [21].

## 3. Localized FCM Clustering

Chen's method can be considered as a hard segmentation method in which each pixel is assigned to an exclusive cluster. However, it is more suitable for medical images that each pixel is given a membership degree of belonging to each cluster, due to the impact of intensity inhomogeneity and noise. In this paper, we introduce the fuzzy belongingness of each pixel into Chen's model and, thus, propose a localized FCM clustering algorithm to implement the task of segmentation and bias field estimation. 

### 3.1. Energy Formulation

Similar to Chen's method, we first consider a task of classifying the data $\tilde{I}(x)$ in the neighborhood *O*_*y*_ into *N* clusters. If the *K*-means clustering in Chen's method is replaced by the FCM clustering, then the objective function in (3) can be converted to the following expression:

(7) $$\begin{array}{c} \varepsilon_{y}=\sum_{i=1}^{N}\int_{O_{y}}u_{i}^{m}\left(x\right)\omega \left(x-y\right){\left|\tilde{I}(x)-\tilde{b}(y)-c_{i}\right|}^{2}dx, \end{array}$$

where *m* > 1 is the fuzzy coefficient, *u*_*i*_(*x*)  (0 ≤ *u*_*i*_(*x*) ≤ 1) is the membership function of pixel *x* belonging to the region *Ω*_*i*_, and *ω*(*x* − *y*) is the same nonnegative weight function as in (3).

Although the choice of the weighting function is flexible, it is preferable to use a weighting function *ω*(*x* − *y*) such that larger weights are assigned to the data $\tilde{I}(x)$ for *x* closer to the center *y* of the neighborhood *O*_*y*_. In this paper, the weighting function *ω* is chosen as a truncated Gaussian kernel

(8) $$\begin{array}{c} \omega \left(d\right)=\{\begin{array}{cc} \frac{1}{a}e^{-{\left|d\right|}^{2}/2\sigma^{2}} & \left|d\right|\leq r,\\ 0 & \text{else}, \end{array} \end{array}$$

where *σ* is the standard deviation of the Gaussian kernel and *a* is a constant to normalize the Gaussian kernel. The above objective function *ε*_*y*_ can be rewritten as follows:

(9) $$\begin{array}{c} \varepsilon_{y}=\sum_{i=1}^{N}\int_{\Omega }u_{i}^{m}\left(x\right)\omega \left(x-y\right){\left|\tilde{I}(x)-\tilde{b}(y)-c_{i}\right|}^{2}dx \end{array}$$

as *ω*(*x* − *y*) = 0 for *x* ∉ *O*_*y*_.

The desired clustering on the entire image domain *Ω* should have a good local performance in terms of the above objective function *ε*_*y*_ for every neighborhood *O*_*y*_. Therefore, we need to minimize *ε*_*y*_ for all *y* ∈ *Ω* like Chen's method [6]. This can be achieved by minimizing the integral of *ε*_*y*_ over *Ω*. As a result, we define the following energy for our proposed localized FCM clustering:

(10) $$\begin{array}{c} \varepsilon =\int_{\Omega }\left(\sum_{i=1}^{N}\int_{\Omega }u_{i}^{m}\left(x\right)\omega \left(x-y\right){\left|\tilde{I}(x)-\tilde{b}(y)-c_{i}\right|}^{2}dx\right)dy. \end{array}$$

### 3.2. Energy Minimization

The above energy *ε* can be minimized in a fashion similar to the standard FCM algorithm. Taking the first derivatives of *ε* with respect to *u*_*i*_(*x*),  $\tilde{b}(y)$, and *c*_*i*_ and setting them to zero results in three necessary but not sufficient conditions for *ε* to be at a local extremum. In this subsection, we will derive these three conditions.

#### 3.2.1. Membership Functions Evaluation

The energy *ε* in (10) is subject to the constraint ∑_*i*=1_^*N*^ *u*_*i*_(*x*) = 1. Thus, this constrained optimization will be solved using one Lagrange multiplier

(11) E=∫Ω(∑i=1N∫Ωuim(x)ω(x−y)|I~(x)−b~(y)−ci|2dx)dy+λ(1−∑i=1Nui(x)).

Taking the derivative of *E* with respect to *u*_*i*_(*x*) and setting the result to zero, we have, for *m* > 1

(12) [∂E∂ui(x)=∫Ωmuim−1(x)ω(x−y)         ×|I~(x)−b~(y)−ci|2dy−λ]ui(x)=ui∗(x)=0.

Solving for *u*_i_*(*x*), we have

(13) $$\begin{array}{c} u_{i}^{*}\left(x\right)={\left(\frac{\lambda }{m\int_{\Omega }\omega \left(x-y\right){\left|\tilde{I}\left(x\right)-\tilde{b}\left(y\right)-c_{i}\right|}^{2}dy}\right)}^{1/\left(m-1\right)}. \end{array}$$

Since ∑_*k*=1_^*N*^ *u*_*k*_(*x*) = 1 for all *x*, we have

(14) $$\begin{array}{c} \sum_{k=1}^{N}{\left(\frac{\lambda }{m\int_{\Omega }\omega \left(x-y\right){\left|\tilde{I}\left(x\right)-\tilde{b}\left(y\right)-c_{k}\right|}^{2}dy}\right)}^{1/\left(m-1\right)}=1 \end{array}$$

or

(15) $$\begin{array}{c} \lambda =\frac{m}{{\left(\sum_{k=1}^{N}{\left(1/\int_{\Omega }\omega \left(x-y\right){\left|\tilde{I}\left(x\right)-\tilde{b}\left(y\right)-c_{k}\right|}^{2}dy\right)}^{1/\left(m-1\right)}\right)}^{m-1}}. \end{array}$$

Substituting (15) into (13), the zero-gradient condition for the membership functions can be rewritten as

(16) $$\begin{array}{c} u_{i}^{*}\left(x\right)=\frac{1}{\sum_{k=1}^{N}{\left(\left(\int_{\Omega }\omega \left(x-y\right){\left|\tilde{I}\left(x\right)-\tilde{b}\left(y\right)-c_{i}\right|}^{2}dy\right)/\left(\int_{\Omega }\omega \left(x-y\right){\left|\tilde{I}\left(x\right)-\tilde{b}\left(y\right)-c_{k}\right|}^{2}dy\right)\right)}^{1/\left(m-1\right)}}. \end{array}$$

#### 3.2.2. Bias Field Estimation

In a similar way, taking the derivative of *E* with respect to $\tilde{b}(y)$ and setting the result to zero, we have

(17) $$\begin{array}{c} {\left[\sum_{i=1}^{N}\int_{\Omega }u_{i}^{m}\left(x\right)\omega \left(x-y\right)\left(\tilde{I}\left(x\right)-\tilde{b}\left(y\right)-c_{i}\right)dx\right]}_{\tilde{b}(y)={\tilde{b}}^{*}(y)}=0. \end{array}$$

Solving for ${\tilde{b}}^{*}(y)$, we have

(18) $$\begin{array}{c} {\tilde{b}}^{*}\left(y\right)=\frac{\sum_{i=1}^{N}\int_{\Omega }u_{i}^{m}\left(x\right)\omega \left(x-y\right)\left(\tilde{I}\left(x\right)-c_{i}\right)dx}{\sum_{i=1}^{N}\int_{\Omega }u_{i}^{m}\left(x\right)\omega \left(x-y\right)dx}. \end{array}$$

#### 3.2.3. Constants *c*_*i*_ Updating

Likewise, taking the derivative of *E* with respect to *c*_*i*_ and setting the result to zero, we have

(19) $$\begin{array}{c} {\left[\iint_{\Omega }^{}u_{i}^{m}\left(x\right)\omega \left(x-y\right)\left(\tilde{I}\left(x\right)-\tilde{b}\left(y\right)-c_{i}\right)dx\;dy\right]}_{c_{i}=c_{i}^{*}}=0. \end{array}$$

Solving for *c*_i_*, we have

(20) $$\begin{array}{c} c_{i}^{*}=\frac{\iint_{\Omega }^{}u_{i}^{m}\left(x\right)\omega \left(x-y\right)\left(\tilde{I}\left(x\right)-\tilde{b}\left(y\right)\right)dx\;dy}{\iint_{\Omega }^{}u_{i}^{m}\left(x\right)\omega \left(x-y\right)dx\;dy}. \end{array}$$

## 4. Exploiting Spatial Information

One of the important characteristics of an image is that neighborhood pixels are highly correlated. In other words, these neighborhood pixels possess similar intensity, and the probability that they belong to the same cluster is great. This spatial relationship is important in clustering, but it is not utilized in a conventional FCM algorithm. To exploit the spatial information, we refer to [15] and define a spatial function as follows:

(21) $$\begin{array}{c} h_{i}\left(x\right)=\sum_{s\in \text{NB}\left(x\right)}u_{i}\left(s\right), \end{array}$$

where NB(*x*) represents a square window centered on pixel *x* in the spatial domain. A 5 × 5 window was used throughout this work. Just like the membership function, the spatial function *h*_*i*_(*x*) represents the probability that pixel *x* belongs to the *i*th cluster. The spatial function of a pixel for a cluster is large if the majority of its neighborhoods belong to the same cluster. The spatial function is incorporated into membership function as follows:

(22) $$\begin{array}{c} u_{i}^{\prime }\left(x\right)=\frac{u_{i}\left(x\right)h_{i}\left(x\right)}{\sum_{k=1}^{N}u_{k}\left(x\right)h_{k}\left(x\right)}. \end{array}$$

**Figure 2:**
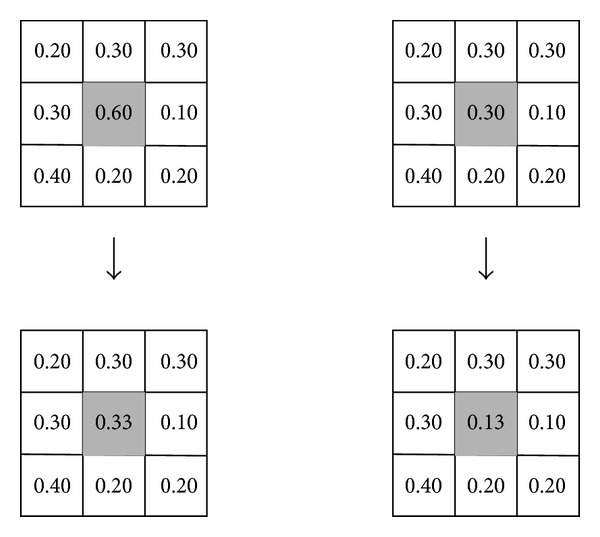
An illustration to demonstrate the effect of removing noise by exploiting spatial information. The left and right columns show the variation of the membership functions of a noisy pixel and a noise-free pixel, respectively.

To demonstrate the effect of removing noise by exploiting spatial information, we use a 3 × 3 neighborhood centered on a pixel under consideration. Without loss of generality, we assume that the image domain is divided into two regions; that is, *N* = 2. Suppose that the values of membership functions of all neighborhood pixels belonging to the first cluster are shown in Figure 2. The upper row corresponds to the original values of membership functions, while the lower row shows the new values of membership functions by using (22). If we set the threshold to 0.50 for defuzzification, then the left and right columns of Figure 2 show the variations of the membership functions of a noisy pixel and a noise-free pixel, respectively. Obviously, the value of membership function of the noisy pixel has a desired correction from 0.60 to 0.33, while the noise-free pixel still belongs to the second cluster with a larger membership function.

In general, the spatial functions simply fortify the original membership in a homogeneous region, and the clustering result remains unchanged. However, for a noisy pixel, (22) reduces the weighting of a noisy cluster by the labels of its neighborhood pixels. As a result, misclassified pixels from noisy regions or spurious blobs can be easily corrected.

## 5. Implementation and Experimental Results

The proposed algorithm is a two-pass process at each iteration. The first pass is to calculate the corresponding variables *u*_*i*_(*x*), $\tilde{b}(y)$, and *c*_*i*_. In the second pass, the membership functions incorporated with the spatial information are updated, and the resulting new membership functions will be inserted into the next iteration. The detailed procedures can be summarized in the following steps.

**Step 1**

Initialize the number of clusters *N*, membership functions *u*_*i*_(*x*), constants *c*_*i*_, and bias field $\tilde{b}(y)$.

**Step 2**

Updating the constants *c*_*i*_ using (20).

**Step 3**

Estimating bias field $\tilde{b}(y)$ using (18).

**Step 4**

Updating membership functions *u*_*i*_(*x*) using (16).

**Step 5**

Computing the new membership functions incorporated with spatial information using (22).

Repeat Steps 2–5 till termination. The iteration is stopped when the maximum difference between constants *c*_*i*_ at two successive iterations is less than a threshold (e.g., 0.001). After the convergence, defuzzification is applied to assign each pixel to a specific cluster for which the membership function is maximal.

In this section, we apply the proposed algorithm to both synthetic and clinical medical images to demonstrate its effectiveness. The parameters used in our algorithm are as follows: fuzzy coefficient *m* = 2, standard deviation of the Gaussian kernel *σ* = 4, and neighborhood radius of the Gaussian kernel *r* = 15. To demonstrate the robustness, the initializations of the variables *u*_*i*_(*x*), $\tilde{b}(y)$, and *c*_*i*_ for the experiments in this paper are all generated as random fields or random numbers.

### 5.1. Segmentation of Synthetic Images

**Figure 3:**
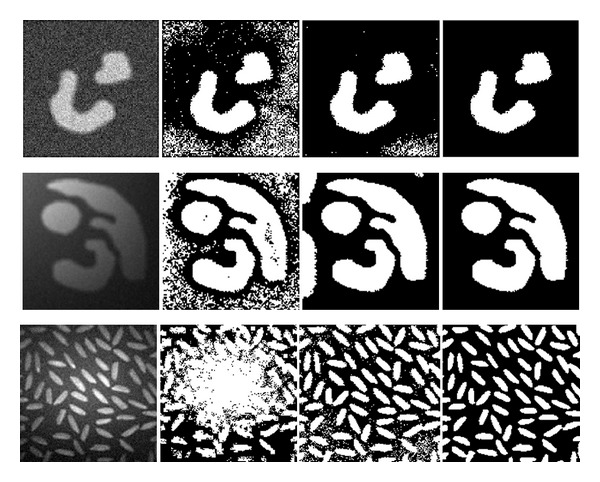
Segmentation results of the proposed algorithm on three synthetic images. Column 1: original images. Column 2-3: intermediate results. Column 4: final results.

The first experiment is performed in three synthetic images, which are displayed in the first column of Figure 3. In the first image, there is strong noise in both object and background regions. The images in the middle and bottom rows are corrupted by noise and intensity inhomogeneity. The intermediate segmentation results obtained by running the proposed algorithm for different numbers of iterations are shown in the second and third columns, and the final results obtained after the convergence of our algorithm are shown in the fourth column. It is revealed from Figure 3 that the result gradually improves during the iterative segmentation process. In the final segmentation results, objects and background can be clearly differentiated despite of the impact of noise and intensity inhomogeneity.

### 5.2. Segmentation of Clinical Medical Images

In this subsection, we compare the proposed algorithm with the bias-corrected FCM (BCFCM) algorithm [12], the sFCM algorithm [15], and the CLIC algorithm [17] for clinical medical images.

**Figure 4:**
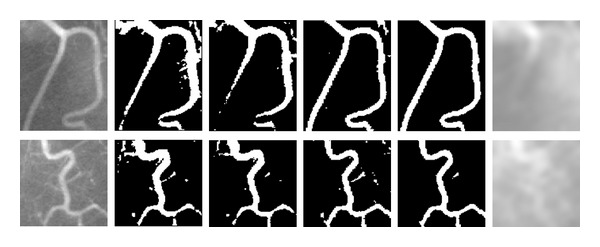
Comparison of segmentation results on two X-ray vessel images. Column 1: original images. Column 2: the BCFCM algorithm. Column 3: the sFCM algorithm. Column 4: the CLIC algorithm. Column 5: the proposed algorithm. Column 6: the estimated bias fields by the proposed algorithm.

The images in the first column of Figure 4 are two X-ray vessel images with noise and intensity inhomogeneity. It can be seen that the upper parts of the images appear brighter, while the lower parts are darker due to the intensity inhomogeneity. As a result, the intensity values of the background in the upper region may be larger than the ones of vessels in the lower region. This phenomenon can cause serious misclassification for those clustering algorithms based on global region. Both of the BCFCM algorithm and the sFCM algorithm are based on global region clustering and hence cannot overcome this problem. This can be observed from the segmentation results which contain some parts of background in the upper brighter region while losing some vessel profiles in the lower darker region. However, the aforementioned phenomenon will become unobvious in local region because intensity inhomogeneity is slowing varying. Therefore, the local region clustering-based algorithm, namely the CLIC algorithm and the proposed algorithm can handle intensity inhomogeneity to obtain the complete vessel profile. Nevertheless, the CLIC algorithm has no step to resist noise so that its results contain some spurious blobs, due to the impact of noise. By contrast, our proposed algorithm utilizes spatial information to suppress noise and thus achieves the desirable segmentation results. The two images shown in the last column of Figure 4 are the estimated bias fields obtained by the proposed algorithm.

**Figure 5:**
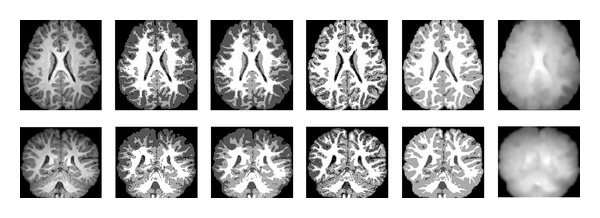
Comparison of segmentation results on two 3T brain MR images. Column 1: original images. Column 2: the BCFCM algorithm. Column 3: the sFCM algorithm. Column 4: the CLIC algorithm. Column 5: the proposed algorithm. Column 6: the estimated bias fields by the proposed algorithm.

We also apply the aforementioned four algorithms to 3T brain MR images. The original images are also corrupted by intensity inhomogeneity and noise, which makes the images brighter in the middle than in the other regions. The task of segmentation is to partition the brain MR images into four regions, that is, white matter (WM), gray matter (GM), cerebral spinal fluid (CSF), and background. The comparison of segmentation results obtained by these four algorithms is shown in Figure 5. Obviously, the BCFCM algorithm and the sFCM algorithm misclassify plentiful WM into GM in the vicinity of the skull because the WM in such region has approximate intensity values with the GM owing to the impact of the intensity inhomogeneity. The segmentation results of the CLIC algorithm show again that it is capable of dealing with the intensity inhomogeneity but unable to suppress the noise. However, our proposed algorithm gets fairly better segmentation with clear and correct classification of tissues. The estimated bias fields obtained by our proposed algorithm are shown in the sixth column of Figure 5.

### 5.3. Quantitative Comparison

**Figure 6:**
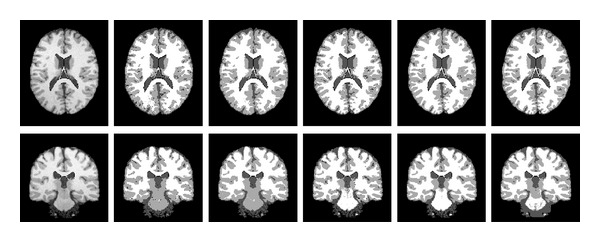
Comparison of segmentation results on two simulated brain MR images. Column 1: original images. Column 2: the BCFCM algorithm. Column 3: the sFCM algorithm. Column 4: the CLIC algorithm. Column 5: the proposed algorithm. Column 6: ground truth.

To quantitatively compare the proposed algorithm with the above-mentioned other three algorithms, we use the T1-weighted simulated brain MR images with ground truth from Brain Web in the link http://www.bic.mni.mcgill.ca/brainweb/. The selected MR images include 40% image intensity inhomogeneity and 3% noise. The original images and the segmentation results are shown in Figure 6. We adopt Jaccard similarity (JS) [19] as a measurement of the segmentation accuracy. The JS between two regions *S*1 and *S*2 is defined as the ratio between the areas of the intersection and the union of them, namely, JS(*S*1, *S*2) = |*S*1∩*S*2|/|*S*1 ∪ *S*2|. We compute the JS between the segmented region *S*1 obtained by the algorithm and the corresponding region *S*2 given by the ground truth. The closer the JS value to 1, the better the segmentation result. The resulting JS values for the four algorithms are listed in Table 1. It can be observed from both Figure 6 and Table 1 that the segmentation results of our proposed algorithm are more accurate than the other three algorithms.

## 6. Discussion

The proposed algorithm suffers from manually setting of two parameters: the neighborhood radius *r* and the standard deviation *σ* of the truncated Gaussian kernel. Note that the radius *r* should be selected appropriately according to the degree of the intensity inhomogeneity. For more localized intensity inhomogeneity, the bias field $\tilde{b}$ varies faster, and therefore the approximation in (4) is valid only in a smaller neighborhood. In this case, a smaller *r* should be used as the radius of the neighborhood, and the standard deviation *σ* should be also selected a smaller value correspondingly. 

**Figure 7:**
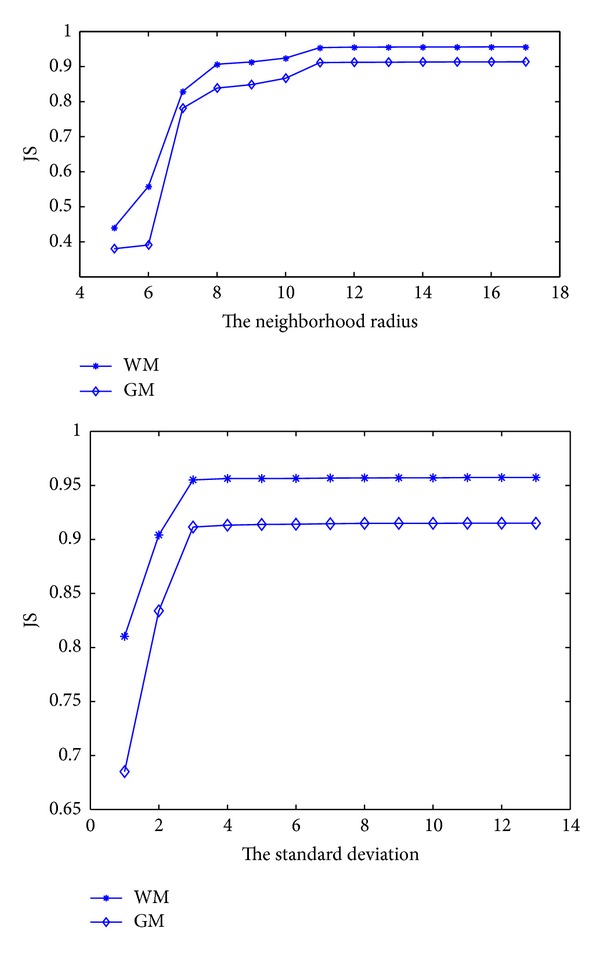
The JS values of the segmentation results obtained by using the different parameters setting of the truncated Gaussian kernel.

Figure 7 shows the JS values of the segmentation results with different parameters selection. The original image is obtained from Brain Web. The upper figure shows the influence of the radius, while the lower figure shows the influence of the standard deviation. The accuracy of segmentations increases with the increasing of *r* and *σ*. When *r* > 10 or *σ* > 3, the JS values of WM and GM increase slightly, while the time consumption would have a significant increase. Considering the segmentation accuracy and the time consumption of the algorithm, we suggest that 9 ≤ *r* ≤ 17 and 3 ≤ *σ* ≤ 6 for this type of image. In our experiments, we set *r* = 15 and *σ* = 4 for all test images.

## 7. Conclusion

In this paper, we have proposed a localized FCM clustering algorithm for simultaneous segmentation and bias field estimation of medical images. The proposed algorithm defines a fuzzy energy that depends on the bias field, membership functions, and the constants that approximate the true signal from the corresponding tissues. Bias field estimation and image segmentation are simultaneously achieved by minimizing this energy. Besides, we also utilize the neighborhood spatial information to resist the noise interference. Moreover, the proposed algorithm is robust to initialization, thereby allowing fully automatic applications. Comparisons with other approaches demonstrate the superior performance of the proposed algorithm.

## Figures and Tables

**Table 1:** Comparison of the JS values of the segmentation results obtained by the four algorithms.

Image	Tissue	BCFCM	sFCM	CLIC	Proposed algorithm
Brain 1	WM	0.8957	0.9139	0.9321	0.9536
	GM	0.8361	0.8598	0.8782	0.9125
	CSF	0.8847	0.8825	0.8902	0.8963

Brain 2	WM	0.8152	0.8201	0.8563	0.8987
	GM	0.7640	0.7716	0.8011	0.8376
	CSF	0.7941	0.8061	0.8128	0.8321
